# Design and content of health facility routine data recording and reporting forms for maternal, newborn, and child health: a scoping review of literature

**DOI:** 10.1093/jamiaopen/ooag096

**Published:** 2026-06-12

**Authors:** Anna Peeler, Stephanie Hammerich-Hille, Moise Muzigaba, Mary Peeler, Yoshiko Sakuma, Nina Gerlach, Ricardo Correia, Theresa Diaz, Elizabeth Katwan, Louise Tina Day

**Affiliations:** Department of Infectious Disease Epidemiology and International Health, London School of Hygiene & Tropical Medicine, London, WC1E 7HT, United Kingdom; Florence Nightingale Faculty of Nursing, Midwifery, and Palliative Care, King’s College London, London, SE1 8WA, United Kingdom; Department of Infectious Disease Epidemiology and International Health, London School of Hygiene & Tropical Medicine, London, WC1E 7HT, United Kingdom; Department of Maternal, Newborn, Child, and Adolescent Health and Ageing, World Health Organization, Geneva, CH-1211, Switzerland; Department of Obstetrics & Gynecology, The University of North Carolina at Chapel Hill, Chapel Hill, NC, 27514, United States; Department of Infectious Disease Epidemiology and International Health, London School of Hygiene & Tropical Medicine, London, WC1E 7HT, United Kingdom; Independent Consultant, Oldenburg, Germany; Faculty of Medicine, University of Porto, Porto, 4050-290, Portugal; Department of Maternal, Newborn, Child, and Adolescent Health and Ageing, World Health Organization, Geneva, CH-1211, Switzerland; Department of Maternal, Newborn, Child, and Adolescent Health and Ageing, World Health Organization, Geneva, CH-1211, Switzerland; Department of Infectious Disease Epidemiology and International Health, London School of Hygiene & Tropical Medicine, London, WC1E 7HT, United Kingdom

**Keywords:** health information systems, data accuracy, data quality, indicators, maternal health, newborn health, child health, user-centered design

## Abstract

**Background:**

High-quality routine health information systems (RHIS) are critical for improving maternal, newborn, and child health (MNCH), to facilitate clinical decision-making and track service delivery. Despite widespread use of health facility data forms for capturing indicators, there are no evidence-based standardized formats or content guidelines for MNCH. The aim of this scoping review was to identify, synthesize, and explore literature related to health facility data forms regarding data-element content, design, and format.

**Methods:**

Following Arksey and O’Malley’s scoping review methodology, we searched 8 databases and grey literature for research related to health facility data forms for MNCH populations. Eligible reports discussed the content (data elements) and/or design (format) of forms. Data were analyzed using thematic synthesis.

**Results:**

We included 54 reports from 47 countries, with most published post-2014. Most reports represented high-mortality MNCH settings and 39% were specific for MNCH content. Structured response formats (e.g., checkboxes and radio buttons) were associated with improved efficiency and data completeness. Few studies involved end users in form design, and only 3 used validated tools to evaluate usability or data quality. One-page, workflow-aligned designs were commonly recommended to reduce cognitive workload.

**Conclusions:**

Standardization of MNCH health facility data-form content and format are currently lacking. User-centered design principles are underutilized. Improving form usability, aligning data capture with clinical workflow, and involving frontline health workers in form development can enhance data quality and reduce documentation burden. Future research should evaluate how form design influences diagnostic accuracy, data quality, and quality of care.

Key findings **What was known before?** 

- Improving the quality of health data for decision-making is a critical component of health system strengthening.
- Paper and digital health facility data forms are used to report data into routine health information systems.
- Currently there are no recommended WHO standardized health facility data forms for maternal, newborn and child health.

**What is new about this study?** 

- Our scoping review identified limited evidence regarding health facility data forms in terms of optimal content and format including very limited instances of evaluation of efficacy, usability, and cognitive workload.
- Design of health facility data forms rarely included active input from the end users, frontline health workers, or benefited from collaboration with a professional designer.
- Efficient formats of health facility data forms can reduce the amount of time and energy required by the end user such as single page, structured design, data elements ordered by workflow to draw the user’s eye to the most important pieces of data.

**What are the implications of all the available evidence?** 

- User-centered focus when designing health facility data forms is critical to optimize efficiency and reduce cognitive workload for frontline health workers and data managers.
- Content of health facility data forms should be purpose-driven (i.e., follow clinical workflow, support sound clinical, and public-health decision-making by the user), linked to data processing functions, and eliminate redundant data elements.
- Data quality assurance mechanisms, including feedback, need to be linked to health facility data forms.

**What are the outstanding research questions?** 

- What is the optimal design to improve efficiency and reduce cognitive workload for frontline health workers in specific contexts?
- What is the association or correlation between content, format, efficiency, and diagnostic accuracy?

## Background

Achieving the 2030 Sustainable Development Goals for maternal, newborn, and child health (MNCH) requires not only expanding access to care but also improving care quality.^1^^,^^2^ Universal provision of high-quality MNCH services could prevent hundreds of thousands of maternal deaths, stillbirths, and millions of neonatal and child deaths annually.^3^^,^^4^ The World Health Organization (WHO) defines quality of care as the extent to which health services are evidence based and improve desired health outcomes and has outlined standards to guide care for MNCH populations.^5–7^ Among these, robust routine health information systems (RHIS) are essential to generate reliable data for clinical decision-making, monitoring service delivery, and guiding health policies.^8^

In many low- and middle-income countries (LMICs), RHIS rely on facility-level data captured by frontline health workers, most often on paper forms before aggregation and upwards reporting.^9^^,^^10^ However, many forms are designed primarily for reporting rather than supporting clinical workflows, and the design process is rarely described in the published literature.^11^ As a result, there is a dearth of evidence-based guidance on how to develop optimal forms and which aspects form content and format impact data quality, accuracy, completeness, timeliness, and usability.^10^^,^^12^ As these forms represent the socio-technical intersection between patient care and data information systems, it is essential that these forms are robust, evidence based, and support optimal workflow for clinicians while feeding high-quality data for monitoring and quality improvement.

Prior WHO guidance emphasizes the importance of high-quality data for decision-making but does not specify which data elements to include or how to structure forms for usability and efficiency.^13^ Efficient, well-designed forms have the potential to reduce the cognitive workload of health workers and free up time for patient care, yet little is known about how design choices influence usability, integration with clinical workflows, digitization, or interoperability across health systems. Therefore, the aim of this scoping review is scoping to identify and synthesize the literature related to health facility data collection, recording and reporting forms regarding data elements (the content of captured data) for MNCH populations and design (format) and to explore their impact on usability, efficiency, data quality, digitization, integration, and interoperability.

## Methods

### Review design

We conducted a scoping review applying Arksey and O’Malley’s methods to identify and synthesize evidence on the design of health data forms and the content of MNCH forms.^14^ A scoping review approach was selected to address the broad and heterogeneous nature of the evidence base on the content, design, and usability of MNCH health facility data forms. Early exploration of the literature indicated substantial variation in study designs, populations, form types (paper and digital), evaluation methods and outcomes, with many reports providing descriptive or developmental accounts rather than evaluative data. Given this diversity, a scoping review was more appropriate than a systematic review, which typically requires narrowly defined questions and a more homogeneous body of evidence to enable formal quality appraisal and synthesis of effect estimates. Likewise, a realist review was deemed less suitable, as the available literature seldom articulated explicit program theories or mechanisms of action that such an approach seeks to test and refine.^15^

We chose Arksey and O’Malley’s methods because they are the most widely used scoping review methodology^16^ and provides a systematic yet flexible approach for mapping the breadth and depth of existing evidence, identifying key concepts, and highlighting gaps in the literature.^14^ This process involved 5 stages: (1) the identification of research question(s), (2) report identification, (3) report selection, (4) data charting, and (5) collating, summarizing, and reporting results. In this review, health facility data forms were defined as documents or tools used as methods for health facility routine data capture and reporting. Hereafter, we refer to them as “forms.” No protocol was registered a priori.

### Stage 1: Identification of research questions

The team identified research questions based on previous literature and commissioning guidance from collaborators in the WHO’s Department of Maternal, Newborn, Child and Adolescent Health and Aging. We sought to answer the following research questions relating to the research aim: (1) How are MNCH data captured in terms of content and format, with a particular focus on LMICs? (2) What processes are used to decide on content to be included in health facility data collection, recording, and reporting forms? (3) What components of design, usability, efficiency, data quality, and digitization are important to consider in the development, implementation, and quality improvement of forms? (4) How are data quality and usability evaluated? (5) What empirical evidence and/or indicators exist for the optimal content and format of MNCH records? (6) How are the integration and interoperability of records implemented and evaluated across the continuum of care? (7) Are digital and paper records designed, implemented, and assessed differently?

These research questions include data-related terms that can be difficult to define. For the purposes of this work, we define the following: Design is the overall structure, layout, and organization of the form^11^; Usability refers to the ease with which healthcare workers can understand, navigate, and complete the form^12^; Data quality refers to the degree to which collected data is accurate, complete, consistent, timely, and reliable^17^; Digitization encompasses the process of moving paper-based forms to electronic or digital formats (i.e., electronic health records and mobile apps)^18^; Integration refers to ability of forms or data collection systems to connect with other health program areas or service delivery workflows; Interoperability refers to the capacity of different digital health systems (i.e., hospital information systems, national reporting platforms, and laboratory systems) to exchange and use data seamlessly^19^; and human-centered design is an approach to creating forms and reporting tools that actively involves the people who will use or are impacted by the data (i.e., healthcare workers, managers, and patients) in the design process.^20^

### Stages 2 and 3: Study identification and selection

We aimed to identify peer-reviewed and gray literature (hereafter “reports”) as improving data is both a research and programmatic issue. Based on input from our multidisciplinary team, a search strategy was developed based on previous literature on MNCH and health facility data forms and included variations of 3 concepts: (1) data collection, recording, reporting forms; (2) elements of design, content, and format; and (3) use by healthcare workers. We searched the following databases: Medline, Embase, Global Health, Global Index Medicus, Cochrane and Web of Science and 3 gray literature databases, Open Access Theses and Dissertations, EBESCO Open Dissertations, and Google (using an incognito browser), with no filters on date published through 28 April 2024 when the search was performed. As no similar reviews have been published and our aim was to understand the full depth and breadth of reports on this topic, we chose not to impose any date restrictions on the search. The search terms are reported in Appendix 1 (see online supplementary material).

Additionally, we hand searched key journals, relevant organizational websites (e.g., WHO, UNICEF, and USAID) and reference lists from related reviews for additional reports as outlined in Arksey and O’Malley’s methods.^14^ All identified reports were uploaded into Covidence. Reports were then screened based on their title and abstract, then retained reports were evaluated for eligibility based on their full text. All reports were screened by at least two team members, and disagreements were discussed by the larger team and adjudicated by consensus. Reports written in other languages with no English language version were translated using ChatGPT for their full texts to be assessed for inclusion. All data were reported using the Preferred Reporting Items for Systematic Reviews and Meta-Analyses (PRISMA) extension for Scoping Reviews guidelines, and the PRISMA checklist can been seen in Appendix 2 (see online supplementary material).^21^

### Inclusion and exclusion criteria

Reports were included if they: (1) focused on health facility data forms and the methods of use for routine data capture and reporting; or (2) discussed content (data elements) of MNCH related variables and/or design (format) of either paper or digital forms. Reports were excluded if they: (1) focused on other aspects of data curation (e.g., data repositories) (2) non-medical records; (3) only focused on data visualization rather than collection, recording, and/or reporting; (4) did not discuss content of MNCH forms or the design or format of forms; (6) were scoping, systematic, or other reviews of literature (i.e., non-primary data); or (7) had no English language title or abstract to screen.

### Stage 4: Data charting

We extracted available salient variables into a Microsoft Excel spreadsheet and organized them in line with the research questions and the guiding methodology (Appendix 3—see online supplementary material). Briefly, variables included the publication source (e.g., journal and grey literature), setting and income classification (World Bank levels^22^), MNCH continuum focus, aims, study design and methods, intervention, form description, form type (register, case note, etc.), content, format, development and evaluation process, usability, efficiency, interoperability considerations, and limitations. Each was extracted from the full text of the reports included by a member of the team and validated by another (AP, SH).

### Stage 5: Collating, summarizing, and reporting results

We conducted narrative synthesis to describe the nature and distribution of the charted data.^23^ To do this, we geographically mapped the included reports, summarized and compared design and content of forms discussed, collated outcome measures used to evaluate forms, and summarized relevant findings. Codes and themes were independently identified by two reviewers (AP, SH) and adjudicated by the wider research team.

## Results

### Search yield

**Figure 1.**
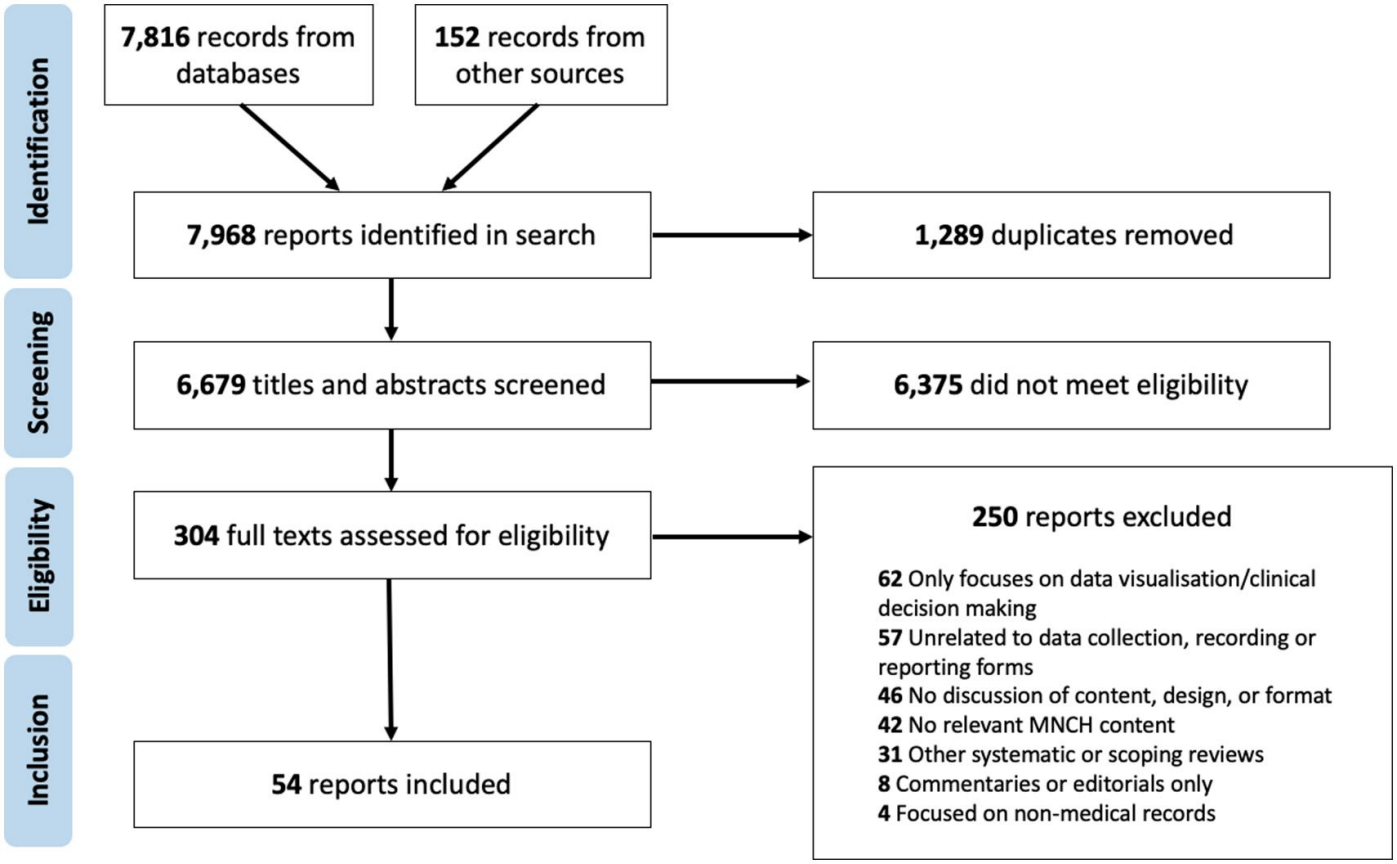
PRISMA flowchart.

We identified 6686 results from the search after deduplication (Figure 1). Using a priori inclusion/exclusion criteria, 6375 were excluded at title and abstract screening for concerning other patient populations, being the wrong type of report (i.e., another review) or otherwise obviously not meeting inclusion criteria. In the full text review, 250 reports were excluded, primarily for only focusing data visualization rather than data recording or reporting (*n* = 59) or not discussing content or design of forms (*n* = 54). In total, *n* = 54 reports were included in the final analysis (Appendix 4—see online supplementary material).^8^^,^^9^^,^^24–75^

Included reports came from several sources, including 40 (74%) from peer-reviewed scientific journals and 8 (15%) from published conference proceedings. Overall, 6 (11%) were from grey literature including other published and unpublished reports or multinational guidelines. Almost two-thirds of the reports (*n* = 31) were published in 2014 or later.

### Overview of included reports

**Figure 2.**
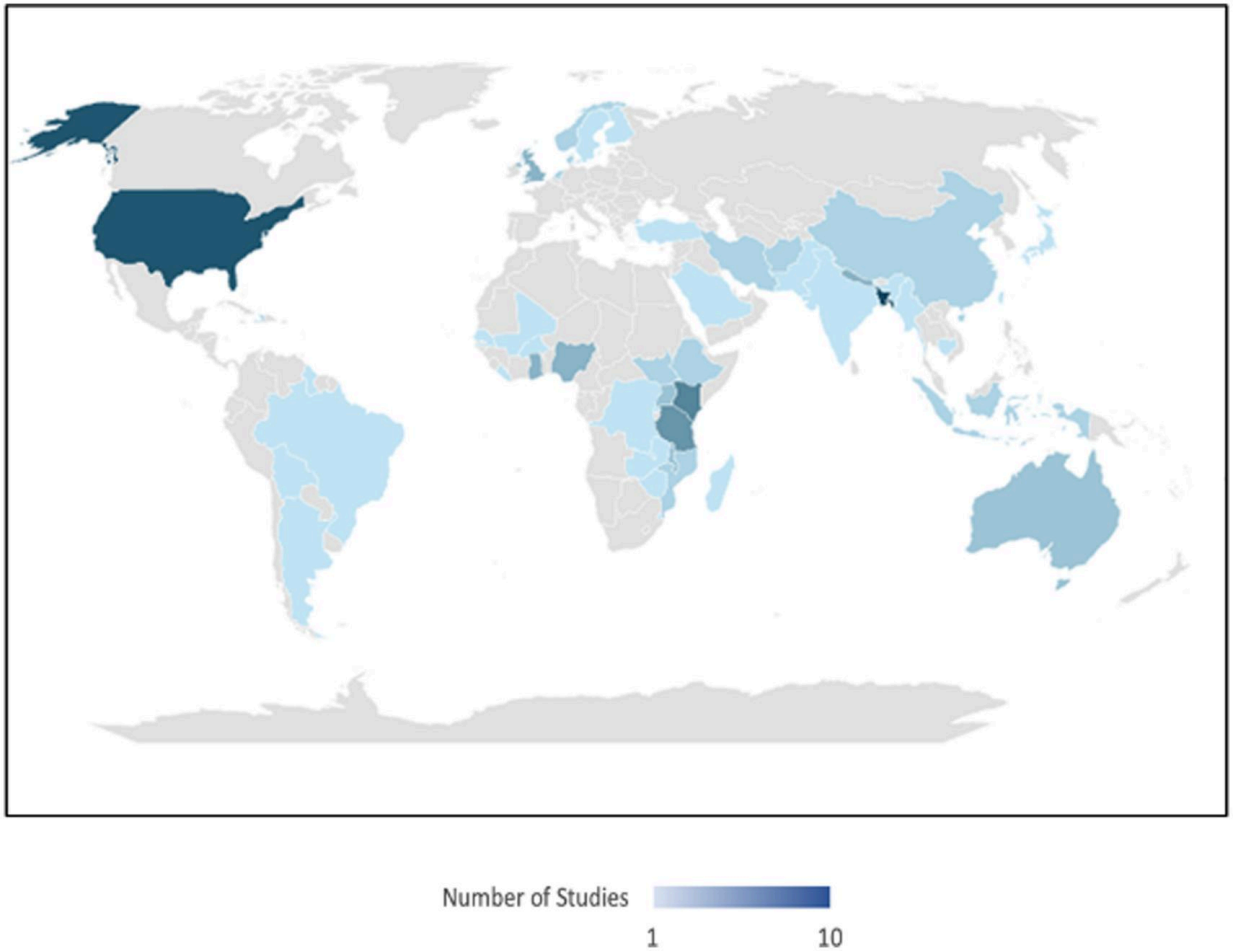
Map of the country settings of included reports on data forms.

Included reports represent work from 47 countries, including 4 reports that were conducted in multiple countries. By WHO regions, the African Region was represented most frequently (*n* = 44), followed by South-East Asia Region (*n* = 20), then the Region of the Americas (*n* = 16), the European Region (*n* = 13), the Eastern Mediterranean Region (*n* = 7) and the Western Pacific Regions (*n* = 7). Based on the 2024 World Bank country income classifications^22^, most reported work that took place in lower-middle income countries (*n* = 55) followed by high-income countries (*n* = 29), low-income countries (*n* = 20), and upper-middle income countries (*n* = 9). Figure 2 shows a map of countries where included reports were conducted.

### Study type

Of the 54 included reports, 36 (67%) were nonexperimental, while 21 (39%) included an experimental component. Of the 36 nonexperimental studies, *n* = 17 were mixed-methods studies regarding the form development, *n* = 13 were descriptive studies of existing forms, *n* = 4 were qualitative studies regarding form development, and *n* = 2 were validation studies. Of the 21 experimental studies, 18 were observational cohort studies and 3 were quasi-experimental. There were no randomized controlled trials included.

### Empirical evidence for optimal content and format of MNCH forms

As mentioned previously in the Methods, in this review, we distinguish between related but analytically distinct constructs: content refers to the data elements included in forms, format and design refer to the structure and layout of those elements.^11^ Usability, efficiency, data quality, integration, and interoperability are evaluation terms that refer to the outcomes and system-level implications associated with how forms are developed and implemented.

#### Content development

As shown in Table 1 and Appendix 6 (see online supplementary material), 19 (35%) reports explored the data elements or content of forms related to 22 different domains of MNCH care. The reports described forms with 7 to 256 data elements. Of those included, 8 reports included details of antenatal care, 8 of intrapartum care, 6 included data on outcomes, 5 on complications, 4 each on sociodemographic variables and lab tests, and 3 on postnatal care. Content of newborn forms included details of inpatient care, discharge diagnoses and outcomes in 2 reports each.

**Table 1.** Content of maternal newborn child health facility forms (*n* = 19 reports)

Domain	Topic	Number of Reports	References
Maternal and newborn	Socio-demographics	4	Danquah^54^, Day^57^, Day^55^, Santos Alves^70^
	Pre-conceptual care	1	Pahlevanynejad^69^
	Antenatal care	8	Hawley^9^, Maternal and Child Survival Program^49^, Bhattacharya^50^, Danquah^54^, Day^57^, Kabue^60^, Pahlevanynejad^69^, Santos Alves^70^
	Intrapartum Care	8	Maternal and Child Survival Program^49^, Bhattacharya^50^, Danquah^54^, Day^57^, Day^55^, Kabue^60^, Kosgei^62^, Pahlevanynejad^69^
	Postnatal care	3	Maternal and Child Survival Program^49^, Bhattacharya^50^, Pahlevanynejad^69^
	Immunization	2	Hawley^9^, Bhattacharya^50^
	Lab tests	4	Hawley^9^, Bhattacharya^50^, Kabue^60^, Santos Alves^70^
	Ultrasound	1	Santos Alves^70^
	Complications	5	Maternal and Child Survival Program^49^, Danquah^54^, Day^57^, Day^55^, Kosgei^62^
	Caesarean section	2	Day^58^, Lodge^64^
	Discharge Diagnoses	1	Day^57^
	Outcomes	6	Maternal and Child Survival Program^49^, Bhattacharya^50^, Danquah^54^, Day^57^, Day^55^, Kabue^60^
	Content not specified	1	Zakaria^47^
Newborn	Socio-demographics	2	Day^56^, Muinga^67^
	Antenatal, Intrapartum care	1	Day^56^
	Newborn inpatient care	2	Tully^46^, Muinga^67^
	Kangaroo Mother Care	1	Save the Children^48^
	Blood tests	1	Day^56^
	Complications	1	Day^56^
	Discharge information	2	Save the Children^48^, Day^56^
	Outcomes	2	Save the Children^48^, Day^56^
Child	IMCI	1	Lugthart^65^

Of those included, 23 (43%) reports discussed the methods of deciding which data elements to include in forms. The most common processes for determining the content of forms were (these categories are not mutually exclusive): adapting previous forms (*n* = 9, 39%); nonsystematic synthesis of expert or research team opinions (*n* = 9, 39%); codesign with key stakeholders (*n* = 8, 35%); reviewing scientific literature (*n* = 7, 30%); human-centered design process (*n* = 2, 9%); and Delphi with experts (*n* = 2, 9%).

In terms of who provided input to the content design of forms, 15 (65%) reported soliciting feedback from multidisciplinary experts, 2 (9%) from single disciplinary experts, 6 (*n* = 26%) from health workers in the setting where the form would be implemented, 6 (*n* = 26%) from researchers or study team members only, and 3 (*n* = 13%) from patient and public involvement groups.

Despite similar populations of interest, no two forms included the same set of data elements or the same combination of domains. While several reports sought to standardize core indicators across countries, the evidence did not converge on a universally accepted set of indicators. This highlights the absence of consensus around “optimal” content for MNCH forms and the tendency to use subjective or nonsystematic methods to develop them.

#### Format

Overall, 21 reports (39%) discussed some aspect of format, including overall form structure, navigation, response options, and actions to complete (Appendix 5.1-5.3—see online supplementary material). Of those, 12 mentioned the length of the form, 15 described the question structure and/or response options, and 13 described some other aspect of the record’s format.

In general, structured response items (i.e., circled text, radio buttons, and checkboxes, drop-down menus) were found to be more efficient (defined by reports as taking less time to complete and with higher accuracy) than unstructured response items (i.e., free text and handwritten notes). Templates and pre-identified response options improved efficiency and data completeness. Checkboxes and radio buttons took the least amount of time and cognitive workload (defined as the mental burden or effort required to complete), followed by drop-down menus, then free text. Tabs to flip between sections or pages rather than one long page that required scrolling was preferred in all 3 reports in which that aspect of format was tested. A summary of key design principles for MNCH forms can be found in Table 2.

**Table 2.** Summary of key design principles for MNCH forms

Format	Content	Development
Simplify forms as much as possible Use low cognitive load question formats and structured responses whenever possible Keep forms to one page when possible to avoid scrolling Bunch items with similar response actions (i.e., multiple choice, short answer, and button) to avoid confusion Draw the user’s eye to the most important pieces of data (i.e., color coding and bolding) Link maternal and newborn data where possible to avoid clinicians having to full in information multiple times	Eliminate redundancies and unnecessary items based on input from end-users and data professionals Order data elements based on established workflows Include opportunities for users to input free text so clinical judgment is not suppressed Include calculating aids (i.e., calculators, instructions for how to calculate scores like APGAR)	User-centred design—include the end-user and data professionals in the design process Forms should be context-specific but have common elements so data can easily be pooled Forms should be linked to data processing functions Include mechanisms for quality assurance and feedback from users

### Evaluation

Of those included, 30 reports (56%) reported at least 1 type of evaluation of forms in terms of usability, efficiency, or data quality. The most commonly evaluated concepts were user satisfaction (*n* = 20), data accuracy (*n* = 15), time to complete (*n* = 12), data completeness (*n* = 12), and usability (*n* = 11), though each report defined these terms in their own way. Most reports used novel or bespoke tools to measure these constructs or evaluated them qualitatively through user feedback. Validated outcome measures were used in just 3 (10%) reports. These measures included the National Aeronautics and Space Administration (NASA) Task Load Index to measure the cognitive workload of filling the form, the Interface Satisfaction Questionnaire and the Software Usability Measurement Inventory to measure user satisfaction, and the Quality of Interactions Schedule.

### Flow of MNCH forms across levels of the health system

Reports indicated that facility-level data were primarily collected for onward reporting to district, regional, and national health information systems, often at the expense of usability for frontline clinical care. In LMICs, monthly summary forms and registers dominated, with health workers often tasked with duplicative documentation across multiple forms. For example, one study noted that primary health facilities used an average of 35 different monthly report forms, taking up to 52 hours per month of staff time.^73^ This reporting burden reflects a tension between meeting national-level data needs and supporting local clinical decision-making.

### Integration and interoperability across the continuum of care

A minority of reports (*n* = 15, 26%) directly addressed integration or interoperability, although this theme is central to health information systems literature. Of those, 6 described efforts to create minimum datasets or archetypes to enable comparability across programs (e.g., premature infant monitoring and prenatal care). Others illustrated how interoperable systems could link maternal and newborn records to reduce duplication and ensure continuity of care.^59^ However, integration was often limited to vertical program reporting (e.g., immunization or antenatal care), rather than horizontal linkage across service delivery points or between facility and community-based systems.

### Differences between paper and digital forms

Comparisons between paper and digital records were rare but instructive.^9^^,^^44^^,^^46^ The 2 studies which compared user preference toward paper vs digital forms found conflicting results: in one, health workers preferred digital data entry, ^44^ while in the other patients and family members preferred paper-based forms.^46^ One report compared the completeness of clinical data collected in paper-based records and digital records and found that digital records demonstrated significant improvements in data completeness and accessibility.^9^

In LMICs, paper registers remained dominant (seen in Appendix 4), with documentation often lengthy and duplicative. Innovations such as structured paper tools (e.g., circle-sheet forms^55–59^^,^^65^ and rubber-stamp templates^61^) improved data completeness and efficiency. In high-income settings, digital records were more prevalent, with evidence that single-page digital forms or tab-based interfaces reduced task time and errors compared to scrolling formats. Studies consistently found that structured digital inputs (radio buttons, dropdowns) enhanced efficiency and accuracy, though free-text fields were still valued for clinical judgment.

## Discussion

This scoping review synthesized evidence on the content, format, design, usability, quality, and interoperability of MNCH paper and digital health facility data forms. Across the included reports, there was no consensus on a standardized or “optimal” data content or format, despite numerous efforts to propose core indicator sets. Consensus on common data elements to be routinely captured for MNCH across diverse settings would be desirable for clinical use, system-level quality improvement initiatives, and routine capture of indicators or decision-making at subnational, national and global use.^60^ Though nearly half of the included reports described the process of developing the forms, only three evaluated usability with validated outcome measures.^38^^,^^44^^,^^69^ Policy decisions to change practice ideally should be based on robust evidence, and evaluations are needed to confirm that newly developed forms are appropriate and effective in capturing prioritized data.

Importantly, the creation of data reporting forms was commonly based on consensus, but this process rarely included users’ or client voices. Though health workers are the primary users of MNCH forms, they were rarely consulted in the development of forms. In the included reports, health facility data were predominantly collected for upward reporting to district and national levels, often at the expense of usability for frontline clinical care. This dual purpose of forms, simultaneously serving reporting needs and clinical workflows, contributed to duplication, inefficiency, and documentation burden. Usability challenges were consistently documented, including excessive cognitive workload, misalignment with clinical workflow, complex layouts, and poor user guidance. Moreover, integration and interoperability across the continuum of care were rarely addressed, with most systems siloed by program area and few examples of alignment with established health information system standards.

Differences between paper and digital records emerged as a central theme. While digital systems provide opportunities for structured input, validation rules, conditional logic, and efficient aggregation, they do not automatically guarantee improvements in usability or data quality. In several studies, poorly designed digital tools introduced new burdens such as excessive navigation, fragmented displays, and misalignment with health worker workflows.^33^^,^^40^^,^^42^ Broader evidence on electronic health record usability supports this finding, emphasizing that interface complexity and poor integration can undermine efficiency and increase error rates.^76^^,^^77^ Conversely, innovations in structured paper tools, such as circle-sheet forms^55–59^^,^^65^ and rubber-stamp templates^61^, demonstrated measurable improvements in data completeness, efficiency, and usability. These findings highlight that digital is not inherently superior to paper; rather, key determinants of success include whether or not the form’s design is aligned with user workflows, minimizes cognitive load, and supports integration of data for both clinical and reporting purposes.

For low-resource settings in particular, these findings carry important implications for implementation and sustainability. Designing effective MNCH data forms requires early and iterative engagement with health workers to ensure forms reflect real workflow constraints.^27^ Adopting a minimal core dataset aligned with national priorities and allowing modular extensions can help reduce form length and duplication.^43^^,^^72^ Consistent design elements, such as logical sequencing, color coding, and structured response options, can reduce mental workload and improve accuracy.^44^^,^^45^ Even so, sustainability depends on adequate training, supervision, and feedback loops that reinforce the value of high-quality data for both clinical and reporting purposes.^33^ Even in paper-first environments, designing with future digitization in mind, such as aligning field names with digital variables and adopting standard codes, can facilitate smoother transitions.^66^

### Future research needs

Future research should move beyond descriptive accounts of form development and content toward empirical evaluation of how specific design choices and design processes (i.e., co-design, human-centered design) affect data quality, usability, and ultimately quality of care. Controlled comparisons of alternative layouts, question structures, and workflow alignments are needed to generate actionable design evidence. Comparative studies of paper and digital records in low-resource MNCH contexts should incorporate validated measures such as the System Usability Scale (SUS) or NASA Task Load Index to quantify usability and cognitive burden. Finally, research should address the equity implications of form design by examining how literacy, language, and gender norms influence data capture and usability in diverse populations.

#### Limitations

There are a few important limitations to this review that should be considered alongside the results. While we sought to undertake comprehensive search of publicly available literature, we were unable to address some sources of selection and publication bias. Many data forms are proprietary to individual health care systems or public health agencies and are not publicly available. As such, we were unable to ascertain the content of some forms beyond what was described in the relevant publication. We also acknowledge that many MNCH data forms used in practice, particularly programmatic tools developed by ministries of health, nongovernmental organizations, or implementing partners, are not publicly available and therefore may not have been retrievable through our search methods. Much of the decision-making and review of forms may be performed internally and not published to be included in this review.

Additionally, although no language restrictions were applied in the search strategy, reports for which English-language titles or abstracts could not be found during the screening phase could not be included, and this may have led to the omission of relevant non-English evidence. We did however screen reports that had English language titles and abstracts but no English language full texts by translating using ChatGPT. However, none of the translated full texts met the other inclusion criteria.

Furthermore, as our inclusion criteria were intentionally broad, we included a number of different reports of varying methodologies which can make drawing conclusions difficult. While we did not conduct a quality assessment of included reports as this is not recommended in the scoping review methodology, many authors of included reports (*n* = 21, 42%) discussed limitations of each of their study designs. The most cited limitations were small sample size or generalizability (*n* = 13, 62% of reports that discussed limitations), measurement limitations (*n* = 8, 38%), time constraints (*n* = 4, 19%), full validation not complete due to pilot/feasibility study (*n* = 3, 14%), and poor data availability (*n* = 2, 9.5%). Other limitations discussed included a lack of comparison group, concerns for contamination of groups, a lack of measurement of patient-centered outcomes, retrospective study design, and social desirability bias. Together, these limitations may affect the generalizability of our findings, as many MNCH data forms used in practice are not publicly available and the included studies were heterogeneous in design and context. Therefore, our conclusions should be interpreted as identifying broadly applicable design principles and evidence gaps rather than universally applicable standards.

## Conclusion

This review highlights that the design and evaluation of MNCH data forms remain fragmented, with substantial heterogeneity in content, persistent usability and efficiency challenges, and limited integration across the continuum of care. Evidence from both paper and digital records shows that well-designed forms that are aligned with workflows, minimize cognitive burden, and incorporate principles of interoperability can improve data completeness, efficiency, and clinical utility. Conversely, poorly designed tools risk creating new burdens and negatively impacting patient care. For clinicians, streamlined forms can reduce documentation workload and enhance decision-making. For policymakers, embedding MNCH forms within national health information systems and aligning them with global standards is critical. And for researchers, rigorous evaluations are needed to establish how specific design choices influence usability, data quality, and quality of care. Strengthening the design and implementation of MNCH records, particularly in low-resource settings, represents an important opportunity to improve data quality, strengthen health systems, and ultimately advance outcomes for mothers, newborns, and children.

## Supplementary Material

ooag096_Supplementary_Data

## Data Availability

This scoping review is based on published literature available through scientific databases and grey literature sources, as described in the Methods and Supplementary Materials. Extracted data elements used in the analysis are provided in the supplementary files. No new or proprietary datasets were generated.
