# Supplementary material for: Design and content of health facility routine data recording and reporting forms for maternal, newborn, and child health: a scoping review of literature
Source: JAMIA Open. 2026 Jun 12;9(3):ooag096. doi: 10.1093/jamiaopen/ooag096 (PMC13264511; doi:10.1093/jamiaopen/ooag096)
Supplement: ooag096_Supplementary_Data [file ooag096_supplementary_data.docx]

Supplementary Material

**Design and content of health facility routine data recording and reporting forms for maternal, newborn, and child health: A scoping review of literature**

Appendix 1. Search Terms

| Concept | Terms |
| --- | --- |
| Clinical routine data collection forms | Data or patient* or medical or inpatient* or outpatient* or health) adj3 (registr* or register* or management or record* or collection or notes or order forms or paper-based systems or information system or text message or digital or internet or online or website or computer* or electronic)) or telemedicine or telemedical or telehealth |
| Design, content, or format | technical* adj3 (Design or Outline or Concept* or Content or format or Modelling or Effective or Efficient or Develop or Tailor or functionality or Integration or interoperability or Implementation or Essence or Requirement or Structure or Context) Data elements or Process-oriented or Data flow organizational change or Data Flow Diagrams or Entity Relationship Diagrams or Class Diagrams or Structure mapping or user-centred design or access to information or communication barrier or common data elements or data accuracy or data aggregation or metadata or pattern recognition, automated |
| Healthcare professionals | Medical or Health or healthcare) adj3 (worker* or force or professional or professionals)) or Healthworker* or Doctor or doctors or GP or GPs or Nurse or nurses or clinician* Health Personnel |

Appendix 2. PRISMA extension for scoping reviews checklist

| **Section and Topic** | **Item #** | **Checklist item** | **Location where item is reported** |
| --- | --- | --- | --- |
| **TITLE** | | | |
| Title | 1 | Identify the report as a systematic review. | 1 |
| **ABSTRACT** | | | |
| Abstract | 2 | See the PRISMA 2020 for Abstracts checklist. | 3 |
| **INTRODUCTION** | | | |
| Rationale | 3 | Describe the rationale for the review in the context of existing knowledge. | 4 |
| Objectives | 4 | Provide an explicit statement of the objective(s) or question(s) the review addresses. | 4 |
| **METHODS** | | | |
| Eligibility criteria | 5 | Specify the inclusion and exclusion criteria for the review and how studies were grouped for the syntheses. | 5 |
| Information sources | 6 | Specify all databases, registers, websites, organisations, reference lists and other sources searched or consulted to identify studies. Specify the date when each source was last searched or consulted. | 5 |
| Search strategy | 7 | Present the full search strategies for all databases, registers and websites, including any filters and limits used. | 5 |
| Selection process | 8 | Specify the methods used to decide whether a study met the inclusion criteria of the review, including how many reviewers screened each record and each report retrieved, whether they worked independently, and if applicable, details of automation tools used in the process. | 5 |
| Data collection process | 9 | Specify the methods used to collect data from reports, including how many reviewers collected data from each report, whether they worked independently, any processes for obtaining or confirming data from study investigators, and if applicable, details of automation tools used in the process. | 5-6 |
| Data items | 10a | List and define all outcomes for which data were sought. Specify whether all results that were compatible with each outcome domain in each study were sought (e.g. for all measures, time points, analyses), and if not, the methods used to decide which results to collect. | 5-6 |
|  | 10b | List and define all other variables for which data were sought (e.g. participant and intervention characteristics, funding sources). Describe any assumptions made about any missing or unclear information. | 5-6 |
| Study risk of bias assessment | 11 | Specify the methods used to assess risk of bias in the included studies, including details of the tool(s) used, how many reviewers assessed each study and whether they worked independently, and if applicable, details of automation tools used in the process. | n/a |
| Effect measures | 12 | Specify for each outcome the effect measure(s) (e.g. risk ratio, mean difference) used in the synthesis or presentation of results. | n/a |
| Synthesis methods | 13a | Describe the processes used to decide which studies were eligible for each synthesis (e.g. tabulating the study intervention characteristics and comparing against the planned groups for each synthesis (item #5)). | 6 |
|  | 13b | Describe any methods required to prepare the data for presentation or synthesis, such as handling of missing summary statistics, or data conversions. | 6 |
|  | 13c | Describe any methods used to tabulate or visually display results of individual studies and syntheses. | 6 |
|  | 13d | Describe any methods used to synthesize results and provide a rationale for the choice(s). If meta-analysis was performed, describe the model(s), method(s) to identify the presence and extent of statistical heterogeneity, and software package(s) used. | 6 |
|  | 13e | Describe any methods used to explore possible causes of heterogeneity among study results (e.g. subgroup analysis, meta-regression). | n/a |
|  | 13f | Describe any sensitivity analyses conducted to assess robustness of the synthesized results. | n/a |
| Reporting bias assessment | 14 | Describe any methods used to assess risk of bias due to missing results in a synthesis (arising from reporting biases). | n/a |
| Certainty assessment | 15 | Describe any methods used to assess certainty (or confidence) in the body of evidence for an outcome. | n/a |
| **RESULTS** | | | |
| Study selection | 16a | Describe the results of the search and selection process, from the number of records identified in the search to the number of studies included in the review, ideally using a flow diagram. | 6 |
|  | 16b | Cite studies that might appear to meet the inclusion criteria, but which were excluded, and explain why they were excluded. | 6 |
| Study characteristics | 17 | Cite each included study and present its characteristics. | Table 1 |
| Risk of bias in studies | 18 | Present assessments of risk of bias for each included study. | n/a |
| Results of individual studies | 19 | For all outcomes, present, for each study: (a) summary statistics for each group (where appropriate) and (b) an effect estimate and its precision (e.g. confidence/credible interval), ideally using structured tables or plots. | Table 1, Table 2 |
| Results of syntheses | 20a | For each synthesis, briefly summarise the characteristics and risk of bias among contributing studies. | 6, 12 |
|  | 20b | Present results of all statistical syntheses conducted. If meta-analysis was done, present for each the summary estimate and its precision (e.g. confidence/credible interval) and measures of statistical heterogeneity. If comparing groups, describe the direction of the effect. | n/a |
|  | 20c | Present results of all investigations of possible causes of heterogeneity among study results. | n/a |
|  | 20d | Present results of all sensitivity analyses conducted to assess the robustness of the synthesized results. | n/a |
| Reporting biases | 21 | Present assessments of risk of bias due to missing results (arising from reporting biases) for each synthesis assessed. | n/a |
| Certainty of evidence | 22 | Present assessments of certainty (or confidence) in the body of evidence for each outcome assessed. | n/a |
| **DISCUSSION** | | | |
| Discussion | 23a | Provide a general interpretation of the results in the context of other evidence. | 24 |
|  | 23b | Discuss any limitations of the evidence included in the review. | 27 |
|  | 23c | Discuss any limitations of the review processes used. | 27 |
|  | 23d | Discuss implications of the results for practice, policy, and future research. | 27 |
| **OTHER INFORMATION** | | | |
| Registration and protocol | 24a | Provide registration information for the review, including register name and registration number, or state that the review was not registered. | 6 |
|  | 24b | Indicate where the review protocol can be accessed, or state that a protocol was not prepared. | 6 |
|  | 24c | Describe and explain any amendments to information provided at registration or in the protocol. | n/a |
| Support | 25 | Describe sources of financial or non-financial support for the review, and the role of the funders or sponsors in the review. | 28 |
| Competing interests | 26 | Declare any competing interests of review authors. | 1 |
| Availability of data, code and other materials | 27 | Report which of the following are publicly available and where they can be found: template data collection forms; data extracted from included studies; data used for all analyses; analytic code; any other materials used in the review. | n/a |

*From:*  Page MJ, McKenzie JE, Bossuyt PM, Boutron I, Hoffmann TC, Mulrow CD, et al. The PRISMA 2020 statement: an updated guideline for reporting systematic reviews. BMJ 2021;372:n71. doi: 10.1136/bmj.n71. This work is licensed under CC BY 4.0. To view a copy of this license, visit <https://creativecommons.org/licenses/by/4.0/>

Appendix 3. Data Extraction Form

| Year published | Study 1 | Study 2 | Study … |
| --- | --- | --- | --- |
| Authors |  |  |  |
| Title |  |  |  |
| Type of Document, Source (i.e., journal, database) |  |  |  |
| Setting |  |  |  |
| MNCH focus |  |  |  |
| Aims, Objectives |  |  |  |
| Study design (if any) |  |  |  |
| Intervention (if any) |  |  |  |
| Description of form |  |  |  |
| Digital or Paper? |  |  |  |
| Content |  |  |  |
| Format |  |  |  |
| Description of form development (if any) |  |  |  |
| Evaluation |  |  |  |
| Outcome measures |  |  |  |
| Findings related to quality, usability, efficiency, or digitization |  |  |  |
| Relevance to high mortality settings |  |  |  |
| Interoperability considerations |  |  |  |
| Limitations |  |  |  |
| Research Question 1 |  |  |  |
| Research Question 2 |  |  |  |
| Research Question 3 |  |  |  |
| Research Question 4 |  |  |  |
| Research Question 5 |  |  |  |
| Research Question 6 |  |  |  |
| Research Question 7 |  |  |  |
| Notes |  |  |  |

Appendix 4. A Descriptive Summary of Included Reports (n=54)

| **Year** | **First Author** | **Title** | **Document Type, Source, Journal** | **Digital or Paper; Setting** | **Aims** |
| --- | --- | --- | --- | --- | --- |
| High Income Countries | | | | | |
| 2015 | Balatsoukas^1^ | User Interface Requirements for Web-Based Integrated Care Pathways: Evidence from the Evaluation of an Online Care Pathway Investigation Tool. | Journal article, Journal of Medical Systems | Digital; UK | To address this gap by evaluating the usability of a novel web-based tool called COCPIT (Collaborative Online Care Pathway Investigation Tool). |
| 1995 | Bearman^2^ | Designing interfaces for medical information management systems. | Conference proceedings, International Medical Informatics Association | Digital; Australia | To summarize the concepts of interface design, discuss the proliferation of poor designs, and will finally illustrate the value of user-centered analysis with a specific example. |
| 2007 | Boye^3^ | User driven, evidence based experimental design; a new method for interface design used to develop an interface for clinical overview of patient records. | Journal article, Studies in Health Technology and Informatics | Digital; Denmark | To develop a method of software development for clinical use that was closer to the conventional way in which technology is matured, validated, and incorporated in everyday activity in the clinical domain. |
| 2013 | Christofidis^4^ | A human factors approach to observation chart design can trump health professionals' prior chart experience | Journal article, Resuscitation | Paper; Australia | To determine whether experienced health workers recognise patient deterioration more accurately and efficiently using (a) novel observation charts, designed from a human factors perspective, or (b) chart designs with which they have long-term experience. |
| 2016 | Chu^5^ | An Efficient User Interface Design for Nursing Information System Based on Integrated Patient Order Information. | Journal article, Studies in Health Technology and Informatics | Digital; Taiwan | To verify the outcome of a new interface design. |
| 2015 | Ehsanullah^6^ | The surgical admissions proforma: Does it make a difference? | Journal article, Annals of medicine and surgery | Paper; UK | To assess whether the quality of documentation was improved when using a standardised surgical clerking proforma compared to freehand clerking at a district general hospital. to assess the attitudes of surgical team members towards the new proforma. |
| 2016 | Froen^7^ | eRegistries: Electronic registries for maternal and child health | Journal article, BMC Pregnancy Childbirth | Digital; Norway | To present frameworks and tools to facilitate the development and secure operation of eRegistries for maternal and child health. |
| 2007 | Haekkinen^8^ | A participatory assessment of IS integration needs in maternity clinics using activity theory | Journal article, International Journal of Medical Informatics | Digital; Finland | To describe the practical problems and solutions of maternity care information management, and to develop and test an easy-to-use method for this purpose. |
| 2011 | Harrington^9^ | Using a unified usability framework to dramatically improve the usability of an EMR Module | Conference abstract, AMIA Annual Symposium proceedings | Digital; USA | To demonstrate how the TURF framework can be used to evaluate the usability of an EMR module and subsequently redesign its interface with dramatically improved usability in a unified, systematic, and principled way. This study also shows how heuristic evaluations can be utilized to complement the TURF framework. |
| 2014 | Hawley^10^ | Sharing of clinical data in a maternity setting: How do paper hand-held records and electronic health records compare for completeness? | Journal article, BMC health services research | Both; Australia | To examine and compare the completeness of clinical data collected in a paper held records PHR and an electronic held records EHR. |
| 2021 | Iftikhar^11^ | Comparing single-page, multipage, and conversational digital forms in health care: Usability study | Journal article, JMIR Human Factors | Digital; UK | To assess the usability of three different interactive forms: a single-page digital form, a multipage digital form, and a conversational digital form (a chatbot). |
| 2018 | Inokuchi^12^ | Interface design dividing physical findings into medical and trauma findings facilitates clinical document entry in the emergency department: A prospective observational study | Journal article, International Journal of Medical Informatics | Digital; Japan | To evaluate the differences in the time to the final documentation entry and the length of emergency department stays using the previous and new systems over time. |
| 2002 | Jaspers^13^ | Cognitive engineering in interface design | Journal article, Studies in Health Technology and Informatics | Digital; Netherlands | To design a new user interface. |
| 2004 | Jaspers^14^ | The think aloud method: a guide to user interface design | Journal article, International Journal of Medical Informatics | Digital; Netherlands | To design a user interface for a pediatric oncologists’ computerized patient record with consideration of their working behavior and of human computer interfacing principles to contribute to oncologists’ efficiency and satisfaction in interaction with the system. |
| 2013 | Koch^15^ | Evaluation of the effect of information integration in displays for ICU nurses on situation awareness and task completion time: A prospective randomized controlled study | Journal article, International Journal of Medical Informatics | Digital; USA | To measure whether nurses’ situation awareness would increase and task completion time decrease when they used an integrated information display compared to traditional displays for medication management, patient awareness and team communication. |
| 2006 | Moller-Jensen^16^ | Measurement of the clinical usability of a configurable EHR | Journal article, Studies in Health Technology and Informatics | Digital; Denmark | To measure the clinical usability of an EHR configured by use of participatory design with clinicians from a neurological stroke unit in order to get input to the County’s future strategy for incremental implementation of EHR. |
| 2013 | Morton^17^ | Validation of the data elements for the health system domain of the PNDS | Journal article, Association of Peri-Operative Registered Nurses Journal | Not specified; USA | To establish the data elements (ie, unambiguous concepts) for the health system domain of the PNDS; define each; and determine whether each proposed element was clear, necessary, measurable, and accurate. |
| 1996 | Poon^18^ | The PEN-Ivory project: exploring user-interface design for the selection of items from large, controlled vocabularies of medicine | Journal article, Journal of the American Medical Informatics Association | Digital; USA | To explore different user-interface designs for structured progress note entry, with a long-term goal of developing design guidelines for user interfaces where users select items from large medical vocabularies. |
| 2007 | Saleem^19^ | Using human factors methods to design a new interface for an electronic medical record | Conference Abstract, AMIA Annual Symposium proceedings. | Digital; USA | To report on two human factors studies designed to provide input and guidance during this reengineering process for a Veterans Health Administration electronic medical record. |
| 2016 | Sarcevic^20^ | Design challenges in converting a paper checklist to digital format for dynamic medical settings | Journal article, Pervasive Computing Technologies for Healthcare | Both; USA | To describe the process of converting a paper-based record to a mobile digital checklist for trauma resuscitation. |
| 2019 | Tully^21^ | Assessing the Potential for Integrating Routine Data Collection on Complementary Feeding to Child Health Visits: A Mixed-Methods Study | Journal article, International Journal of Environmental Research and Public Health | Both; UK | To assess items for inclusion in routine data collection, their suitability for collecting informative data, and acceptability among health visitors in the UK |
| 2020 | Ward^22^ | Design and Rationale for Common Data Elements for Clinical Research in Pediatric Critical Care Medicine. | Journal article, Pediatric Critical Care Medicine | Not specified; USA | To establish a road map for the development of multinational, multidisciplinary consensus based common data elements that could be adapted for use within any pediatric critical care subject area |
| 2018 | Wilbanks^23^ | The effect of data-entry template design and anesthesia provider workload on documentation accuracy, documentation efficiency, and user-satisfaction. | Journal article, International Journal of Medical Informatics | Digital; USA | To explore the impact of data-entry template design and anesthesia provider workload on documentation accuracy, documentation efficiency, and user-satisfaction to identify the most beneficial data-entry methods for use in future documentation interface design. |
| 2021 | Wilbanks^24^ | Impact of Data Entry Interface Design on Cognitive Workload, Documentation Correctness, and Documentation Efficiency. | Conference abstract, AMIA Joint Summits on Translational Science proceedings | Digital; USA | To evaluate how pairing specific computer-assisted data entry types to specific anesthesia documentation data elements influenced documentation correctness, documentation efficiency, and cognitive workload of anesthesia providers. |
| 2020 | Zakaria^25^ | Development and usability testing of Riyadh Mother and Baby Multi-center cohort study registry. | Journal article, Journal of infection and public health | Digital; Saudi Arabia | To test the usability of the cohort registry of mothers and newborns developed for clinical research and service improvement. |
| Low- and Middle-Income Countries | | | | | |
| 2018^26^ | Maternal and Child Survival Program (USAID) | What Data on Maternal and Newborn Health Do National Health Management Information Systems Include? A review of data elements for 24 low and lower middle-income countries | Technical Report, expert referral | Both  Afghanistan, Bangladesh, Democratic Republic of Congo, Ethiopia, Ghana, Haiti, India, Indonesia, Kenya, Liberia, Madagascar, Malawi, Mali, Mozambique, Myanmar, Nepal, Nigeria, Pakistan, Rwanda, Senegal, South Sudan, Tanzania, Uganda, Zambia | To systematically document key maternal and newborn data elements found in HMIS documents at facility and subnational levels in the USAID priority maternal and child health countries. |
| 2018^27^ | Save the Children | Improving availability and quality of routine data for newborns: Malawi's experience with Kangaroo Mother Care (KMC) | Policy brief, grey literature database | Not specified; Malawi | To develop a national routine reporting system for KMC, including a simplified, user-friendly KMC register and reporting form. |
| 2021 | Adane^28^ | Exploring data quality and use of the routine health information system in Ethiopia: a mixed-methods study. | Journal article, BMJ | Both; Ethiopia | To analyse data quality at the district and regional level and explore factors and perceptions affecting the quality and use of routine data. |
| 2020 | Bhattacharya^29^ | Improving the quality of routine maternal and newborn data captured in primary health facilities in Gombe State, Northeastern Nigeria: a before and after study | PhD dissertation, grey literature database | Both; Nigeria | To assess nine data quality metrics for 14 maternal and newborn health data elements, following implementation of an integrated, district-focused data quality intervention. |
| 2023 | Birabwa^30^ | Quality of routine data related to facility-based maternal mortality measurement in Kampala City, Uganda | Preprint, grey literature database | Digital; Uganda | To assess the quality of routine data on deliveries, livebirths and maternal deaths in Kampala City, Uganda. |
| 2012 | Burkle^31^ | Emergency Surgery Data and Documentation Reporting Forms for Sudden-Onset Humanitarian Crises, Natural Disasters and the Existing Burden of Surgical Disease | Journal article, Prehospital Disaster Medicine | Not Specified; Haiti | To develop a utilitarian and universal form for reporting and data documentation for surgical care during humanitarian crises and natural disasters. |
| 1997 | Danquah^32^ | Improving recordkeeping for maternal mortality programs, Kumasi, Ghana | Journal article, International Journal of Gynecology and Obstetrics | Paper; Ghana | To revise registers to collect information on complications and time of treatment. |
| 2009 | Day^33^ | Structured Neonatal Clinical Sheets—Design and Implementation of a Useful Clinical Tool | Conference abstract, expert referral | Paper; Bangladesh | To observe whether the implementation of structured sheets in the neonatal case-records is a useful tool in the busy neonatal unit of a rural comprehensive emergency obstetric care facility in north-west Bangladesh. |
| 2009 | Day^34^ | Structured Obstetrical Clinical Sheets – the design and implementation of a useful clinical tool | Conference abstract, expert referral | Paper; Bangladesh | To observe whether the implementation of structured sheets in the obstetric case-records is a useful tool in a busy, rural, comprehensive emergency obstetric unit in north-west Bangladesh. |
| 2010 | Day^35^ | Strengthening Caesarean section services - A case study from a rural integrated health and development project in Bangladesh | Conference presentation, expert referral | Paper; Bangladesh | To describe an integrated approach to providing quality, appropriate and timely caesarean section services |
| 2011 | Day^36^ | Implementation of an Integrated Hospital Information System in limited-resource Setting in Rural Bangladesh | Conference abstract, expert referral | Both; Bangladesh | To describe programmatic experience in the rural hospital setting of the implementation of an integrated hospital information system— including the monitoring of indicators of quality of care in the context of serving the poor. |
| 2020 | Day^37^ | Labour and delivery ward register data availability, quality, and utility - Every Newborn - birth indicators research tracking in hospitals (EN-BIRTH) study baseline analysis in three countries | Journal article, BMC Health Service Research | Paper  Bangladesh, Nepal, Tanzania | To assess the availability, quality, and utility of routine labour and delivery ward register data. |
| 2015 | Haskew^38^ | Implementation of a cloud-based electronic medical record for maternal and child health in rural Kenya | Journal article, International Journal of Medical Informatics | Digital; Kenya | To evaluate the impact of a novel cloud-based electronic medical record system on improving completeness of data collected by clinical and public health services. |
| 2023 | Kabue^39^ | Availability of priority maternal and newborn health indicators: Cross-sectional analysis of pregnancy, childbirth and postnatal care registers from 21 countries | Report, grey literature database | Both  Afghanistan, Argentina, Bolivia, Burkina Faso, Eswatini, Ghana, Guyana, Indonesia, Kenya, Malawi, Nepal, Nigeria, Norway, South Sudan, Sweden, Tanzania, Uganda, USA, Palestine, Zimbabwe, Zambia | To report on an analysis of key data elements in ANC, childbirth and PNC registers from 21 countries describes differences in the way data elements are captured, and highlights opportunities for inclusion of some critical data elements that are not currently collected in registers as well as possibly removing some data elements to reduce redundancy across the different registers and other data collection documents. |
| 2018 | Kleczka^40^ | Rubber stamp templates for improving clinical documentation: A paper-based, m-Health approach for quality improvement in low-resource settings | Journal article, International Journal of Medical Informatics | Paper; Kenya | To examine the effect of using rubber stamp templates on clinical documentation in paper-based charts. |
| 2016 | Kosgei^41^ | Quality Of Comprehensive Emergency Obstetric Care Through the Lens of Clinical Documentation on Admission to Labour Ward | Journal article, East African Medical Journal | No specified; Kenya | To determine the level of quality of comprehensive emergency obstetric care, through the lens of clinical documentation of process indicators of selected emergency obstetric conditions that mostly cause maternal mortality on admission to labour ward. |
| 2010 | Liu^42^ | Harmonization of health data at national level: a pilot study in China | Journal article, International Journal of Medical Informatics | Digital; China | To collect health data items that are now nationally available in  various health information systems and harmonize them by modelling and defining the data elements. |
| 2020 | Lodge^43^ | Assessing completeness of patient medical records of surgical and obstetric patients in Northern Tanzania | Journal article, Global Health Action | Paper; Tanzania | To examine surgical and obstetric patient medical record data quality in health facilities as part of a surgical system strengthening initiative in northern Tanzania. |
| 2011 | Lugthart^44^ | Adherence to WHO/UNICEF guidelines of Integrated Management of Childhood Illness with use of adapted circle forms at LAMB, Bangladesh | Masters thesis, expert referral | Paper; Bangladesh | To examine the adherence to and experiences with the IMCI-guidelines by health workers of LAMB, comparing the standard recording forms with the adapted circle forms. |
| 2022 | Muinga^45^ | Implementing a comprehensive newborn monitoring chart: Barriers, enablers, and opportunities | Journal article, PLOS Global Public Health | Paper; Kenya | To report on a process of implementing a comprehensive newborn monitoring chart and the perceptions of health workers in a network of hospitals in Kenya. |
| 2021 | Muinga^46^ | Using a human-centered design approach to develop a comprehensive newborn monitoring chart for inpatient care in Kenya | Journal article, BMC Health Services Research | Paper; Kenya | To improve the documentation of newborn care within a network of hospitals in Kenya, involving nurses and other health workers and applying an adapted Human-Centered Design approach to the design process. |
| 2006 | Mwakyusa^47^ | Implementation of a structured paediatric admission record for district hospitals in Kenya--results of a pilot study | Journal article, BMC Int Health Hum Rights | Paper; Kenya | To examine the feasibility and acceptability of a structured paediatric admission record (PAR) for district hospitals as a means of improving documentation of illness. |
| 2022 | Pahlevanynejad^48^ | Design, implementation, and evaluation of an innovative intelligence information management system for premature infants | Journal article, Digital Health | Not specified; Iran | To design, implement and evaluate an innovative intelligence information management system for premature infants. |
| 2015 | Santos Alves^49^ | Validation of Minimum Data of Archetyped Telehealth Clinical Report for Monitoring Prenatal Care | Journal article, Studies in Health Technology and Informatics | Digital; Brazil | To validate the minimum data set that should be used in the construction of medical records of archetyped telehealth for prenatal care. |
| 2018 | Shahraki^50^ | Smart Acute Stroke Quality Registry Design-Data Elements Identification. | Journal article, Journal of Registry Management | Paper; Iran | To identify appropriate data elements for a national stroke registry to be used to quality. |
| 2021 | Shamba^51^ | Barriers and enablers to routine register data collection for newborns and mothers: EN-BIRTH multi-country validation study | Journal article, BMC Pregnancy Childbirth | Paper  Bangladesh, Nepal, Tanzania | To assess measurement validity for selected Every Newborn coverage indicators. |
| 2021 | Siyam^52^ | The burden of recording and reporting health data in primary health care facilities in five low- and lower-middle income countries | Journal article, BMC Health Services Research | Paper  Cambodia, Ghana, Mozambique, Nigeria, Tanzania | To document the numbers of registers and reporting forms use at the PHC level and to estimate the time it requires for health workers to meet data demands. |
| 2008 | Tasa^53^ | A case study on better iconographic design in electronic medical records' user interface. | Journal article, Informatics for Health & Social Care | Digital; Turkey | To suggest, from the ‘design art’ perspective, a method for improving the usability of an electronic medical record (EMR) interface. |
| 2019 | Zhang^54^ | On standardisation of basic datasets of electronic medical records in traditional Chinese medicine. | Journal article, Computer methods and programs in biomedicine | Digital; China | To standardise a basic dataset of electronic medical records in traditional Chinese medicine |

Abbreviations – ANC: antenatal care; EHR: electronic health record; EMR: electronic medical record; KMC: kangaroo mother care; PHC: primary health care; PNC: postnatal care; UK: United Kingdom; USA: United States of America; USAID: United States Agency for International Development.

*Reports organized by technical reports, then peer-reviewed reports in alphabetical order

Appendix 5.1 Format of paper health facility routine data recording and reporting forms (n=10)

| **Year** | **First author** | **Length of form** | **Format of entry fields** | **Other format considerations** | **Findings related to format design** |
| --- | --- | --- | --- | --- | --- |
| **Appendix 5.1 Paper Forms** | | | | | |
| **Appendix 5.1.1. Single entry format** | | | | | |
| 2018 | Kleczka | One page per rubber stamp | - Bubble format data entry | - Rubber stamps were used to print templates into paper charts, providing clinicians with checklists for use during consultations. - The intervention also included booklets of guidelines and one Android phone for digitizing images of templates. | 1) Documentation completeness increased with the use of the templates.  2) Bubble format data can be digitized from images taken on mobile phones. |
| 2021 | Muinga | One page | - Standardise action to complete items to prevent any confusion - Multiple choice items were designed to have as few options as possible. | - Chart was designed to mimic workflow - Standardised units were used to prevent errors. - Common knowledge abbreviations only. - A color-coded information sheet with normal and out-of-range values for vital signs was included for less experienced users. | 1) Users generally found the forms easy to use.  2) Some gave suggestions to improve the form, and these were incorporated into subsequent drafts. |
| 2006 | Mwakyusa | Not specified | - Circle options - Free text | - The record was redesigned to eliminate duplicate or unnecessary questions, significantly shortening it. | 1) Use of form increased from 50% to 84%.  2) The quality of documentation also improved considerably over time in that important clinical features were documented more completely.  3) Most symptoms and signs can be evaluated rapidly, and the newly designed record permitted rapid documentation (circling an option). |
| 2021 | Siyam | Average of 25 cells to fill for each patient | - All forms were tables with cells as blanks to input data. | None | 1) It took between 2 and 5 minutes to fill in the register for each patient (between 24 and 50% of the consultation time).  2) More cells meant more time to complete register (1 min/6 cells).  3) Health facilities had an average of 35 different monthly report forms to fill out. It took between 10 and 52 hours to complete these forms each month. |
| **Appendix 5.1.2 Multiple entry format** | | | | | |
| 2009 | Day | Each form is one page | - Hybrid circle sheet tools - used as the routine medical by doctors as well as for database data entry by data assistants. - Circle pre-printed options - Every line needs an answer | - Data entry forms double as data visualization for clinical data use - Vertical dark line divides the sheet in two. - To the left of the line are normal and near normal answers (low risk) - To the right of the line are abnormal clinical findings (high risk); further right means more critical (with color coding for most critical) | Resulted in improvements in several areas:  1) All areas of clinical documentation became more detailed and complete.  2) Efficiency increased as circling takes less time than long-handwriting; pertinent findings can be clearly and easily identified as the high-risk information is on the right of the page.  3) Printed options acts as clinical prompts for new and junior  4) For data entry and reporting, variables can easily be entered into customised database with validations, meaning no summary forms or data aggregation tasks are needed. |
| 2009 | Day | Each form is one page | - Hybrid circle sheet tools - used as the routine medical by interprofessional team of midwives and doctors as well as for database data entry by data assistants. - Circle pre-printed options - Every line needs an answer | - Data entry forms double as data visualization for clinical data use - Vertical dark line divides the sheet in two. - To the left of the line are normal and near normal answers (low risk) - To the right of the line are abnormal clinical findings (high risk); further right means more critical (with color coding for most critical) | Resulted in improvements in several areas:  1) Improvements in clinical data/information completeness,  2) Increased documentation efficiency - circle pre-printed answers instead of longhand writing  3) Basic teaching job aid for new and junior midwives and doctors - the printed options acting as clinical prompts.  4) Direct data entry from the clinical sheet of selected variables entered to customised database with validations, by data assistants in the Management Information Systems-Research department (MIS-R). |
| 2010 | Day | Each form is one page | - Circle pre-printed options with option for free text when needed - Every line needs an answer | - Data entry forms double as data visualization for clinical data use - Vertical dark line divides the sheet in two. - To the left of the line are normal and near normal answers (low risk)   - To the right of the line are abnormal clinical findings (high risk); further right means more critical (with color coding for most critical) | 1) Aids for clinical procedure teaching  2) Facilitates clinical decision-making with clinical prompts  3) Saves documentation time by circling instead of long handwriting  4) Improves interprofessional communication  5) Improve clinical information/ data for quality improvement and clinical audit |
| 2011 | Day | Register entry and one-page hybrid form corresponds to matching linked page on database | Hybrid circle pre-printed options correlate to drop-down or radio button on database. | - Registered are simplified as much as possible – duplicates and parallel structures are removed. - Mother and newborn linked | Interoperable system between 1) source data collection forms (registers and individual hybrid medical records) with customized database 2) team of health workers capturing data with Management Information System-Research Department team. Ongoing since 2008 the “Flow Information System Hospital (FISH)” enables efficient provision of all activity-reporting needs of clinical staff (quality improvement, clinical audit), hospital managers (human and physical resource planning), government reporting, and donor partners. |
| 2011 | Lugthart | Each form is one page | - Circle pre-printed options with option for free text when needed - Every line needs an answer | - Data entry forms double as data visualization with clinical assessment and classification arranged in IMCI “traffic light” system.   - left of the line are normal (green) and near normal   - middle are moderately abnormal (yellow)   - right severely abnormal. (red) | Comparing the standard form to the adapted circle-sheet form:  1) Danger signs checked: standard form 73%, adapted form 100%, p=0.03.  2) Time to fill form: standard 19 minutes, adapted form 21 minutes.  3) More mistakes made using standard form compared to adapted form including weight comparison (OR 5.58, p=0.004).  4) Most health workers preferred the adapted form due to less time spent writing, no need to use the chart booklet for classification, and a better overview of the form. FGDs positively identified saving time of health workers, improving feeding assessments, focusing health teaching, and ensuring completeness of assessment and appropriate use of medicine. |
| 2021 | Shamba | Between 35 and 58 columns | - In Bangladesh register columns were ticked when the intervention/practice was done and left blank when not done; in Tanzania, register columns were filled with yes/no. | - One data element was captured per column in the register in Tanzania, but more than one in some register columns in Bangladesh and Nepal. | 1) Forms varied widely between facilities.  2) Often completeness of forms was valued more highly than accuracy.  3) Registers had complex design elements which made completing vital data elements more challenging.  4) Register filling was performed by over-stretched nurse-midwives with variable training, limited supervision, and availability of logistical resources.  5) Use of data from registers was limited due to the lack of trust in its quality. |

Appendix 5.2 Format of digital health facility routine data recording and reporting forms (n=8)

| **Year** | **First author** | **Length of form** | **Format of entry fields** | **Other format considerations** | **Findings related to format design** |
| --- | --- | --- | --- | --- | --- |
| **Appendix 5.2 Digital Forms** | | | | | |
| **Appendix 5.2.1 Single entry format** | | | | | |
| 2016 | Chu | Not specified | A new user interface is compared to the old in this study:   - Old: Pull-down menu - New: Check boxes | None | 1) Time for data entry was reduced from 22.8 sec/record to 3.2 sec/record.  2) Data entry procedures reduced from 9 steps to 3 steps with the new checkbox form.  3) Completeness increased from 20.2% to 98%. |
| 2008 | Tasa | Not specified | None | - Icons, rather than text, are used to represent concepts | 1) In the final version of form, 7.53 icons out of 10 were matched correctly.  2) All icons except three were matched correctly in 83.3% of the forms. |
| **Appendix 5.2.2 Multiple entry format** | | | | | |
| 2021 | Iftikhar | Three forms:  1. single-page form  2. multipage form  3. conversation form (chatbot) | - Items included a combination of select all the apply, radio buttons, drop down items, and free text entry. | - Sections and workflow were structured using human factors design principles to improve usability. | 1) the digital single-page form outperformed the other two forms in almost all usability metrics (lowest task completion time).  2) post-experiment questionnaire indicated that the single-page form was the preferred choice. |
| 1996 | Poon | Multiple prototypes were tested comparing:  1. scrolling page  2. Flipping between pages | None | Multiple prototypes were tested comparing:  1. Dynamic vs Fixed Palette  2. Display of all findings vs Subset of findings | The prototype that allowed the fastest data entry had a paging rather than a scrolling form, used a fixed palette of modifiers rather than a dynamic “pop-up” palette, and it made available all findings from the controlled vocabulary at once rather than displaying only a subset of findings generated by analyzing the patient’s problem list. |
| 2007 | Saleem | The interface was changed from a central dashboard where you could click on any form to one consolidated form where you can switch between tabs. | - Patient reminders include radio buttons to indicate actions taken, and clear, more info, next, back, finish, and cancel buttons to navigate between reminders. | - Clinical reminders were prefaced with a ‘P’ or ‘N’ for primary care provider or nurse to clarify who was responsible for attending to the reminder | The newly designed electronic case note took statistically significantly less time to complete than the old version, meaning it was more usable. |
| 2016 | Sarcevic | Multiple pages were included on thematic tabs | - Complete items are grey; incomplete items are black - For optional items, "N/A" was automatically checked but could be unchecked - For values that indicate actions, the action box for is automatically checked when value is entered | - Tab had number of incomplete items at top - Users could take handwritten notes on each item if needed by tapping a white rectangle next to the item. Notes could be edited later. - For numeric items, a numeric keyboard pops up when the user selected the item - Timestamps are recorded to help with quality assurance. | Grouping checklist items into tabs improved efficiency and prevented scrolling. Handwritten note taking linked to individual items allows for quick jotting of notes but makes using the data later more difficult (as handwriting is often not easily read by computer programs). |
| 2021 | Wilbanks | Not specified | - Forms included a combination of drop-down boxes, radio buttons, checkboxes, and free text with autocomplete suggestions. | None | 1) Checkboxes had worst documentation correctness; radio buttons had best.  2) A higher number of mouse clicks occurred with the use of radio buttons and free text and resulted in the highest documentation correctness.  3) The total time spent documenting from the most to least efficient is check-boxes (10.66 seconds), radio buttons (11.57 seconds), drop-boxes (16.11 seconds), and free text (30.65 seconds).  4) Free text had the highest cognitive workload. |
| 2020 | Zakaria | 13 forms with a total of 256 fields | - Questions are a combination of free text, drop down boxes, and radio buttons. - The system validates entered data as being the correct range and type (numerical vs. text). - Users can export data into text (csv) or spreadsheet (Excel) format for data analysis purposes. | - The user can navigate from one form to another using the quick link menu. - The system has a function that highlights any empty required fields in grey to guide users. - A "not applicable” option was added so users could fill in required fields even when they were not ready to answer the questions. - A calculator for expected delivery date and body mass index was added. | 1) The registry was found to be efficient, easy to learn, satisfactory, and easy to remember, and resulted in fewer errors.  2) A few design suggestions were discussed during the focus group and improvements were made where necessary. For example, font size of logout button was enlarged so it would be more visible to users. |

Appendix 5.3 Format of hybrid or unspecified health facility routine data recording and reporting forms (n=3)

| **Year** | **First author** | **Length of form** | **Format of entry fields** | **Other format considerations** | **Findings related to format design** |
| --- | --- | --- | --- | --- | --- |
| **Appendix 5.3 Digital and Paper Forms OR Not Specified** | | | | | |
| **Appendix 5.3.1 Multiple entry format** | | | | | |
| 2012 | Burkle | One page | - Case note form includes a combination of free text demographic information, dates to indicate when actions were performed, circle items, and check boxes wherever possible to speed documentation. - Reporting form includes numerical answers for demographics and common surgical procedures. | - Data element categories/sections are bolded to visually break up the page. | The purpose of the standard data is to:  1) ensure that all surgical providers, especially from indigenous first responder teams and others performing emergency surgery, from national and international medical teams, contribute relevant and purposeful reporting.  2) provide universally acceptable forms that meet the minimal needs of both national authorities and the Health Cluster.  3) increase transparency and accountability, contributing to improved humanitarian coordination.  4) facilitate a comprehensive review of services provided to those affected by the crisis. |
| 2019 | Tully | Two pages | - Each question has a corresponding blank where the health visitor can enter the value. - Some questions have two blanks to indicate how many times per day OR week the child eats a particular kind of food. | - All answers except one require numerical values. - One additional tick box question at the end indicates if the parent mentioned "baby-led weaning" | 1) Many parents felt more comfortable with paper forms because they felt the digital forms were prone to technical issues and made them feel like they were being tested.  2) The two blanks for different time recall were confusing to parents. This was changed to one time point in the final version. |
| 2018 | Wilbanks | Not specified | - The first site utilized electronic documentation that relied on auto-filling of unstructured data with concurrent documentation of event time and additional narrative text. - The second site used computer-assisted data collection that did not force the documentation of the time of occurrence at the same time as the narrative portion; incorporated a context-sensitive display of documentation options that was customized to the anaesthesia workflow. - The third site used traditional, manually completed paper-based documentation. | None | 1) Auto-filling (61.2%) had the lowest documentation accuracy scores compared to computer-assisted (81.3%) and paper-based documentation (76.2%).  2) Computer-assisted data-entry had the best documentation efficiency scores and required the least percentage of the nurse anesthetists’ time (9.65%) compared to auto-filling (11.43%) and paper-based documentation (15.23%).  3) Paper-based documentation had the highest perceived workload scores compared to auto filling and computer assisted data-entry. |

Appendix 6. Content of MNCH Forms

| **Year** | **First author** | **MNCH domain** | **Type of document** | **Digital or paper** | **Number of data elements** | **Topics included** |
| --- | --- | --- | --- | --- | --- | --- |
| 2018 | Maternal and Child Survival Program^55^ | Maternal, newborn | Registers, reporting forms | Both | Varied between service areas and countries | 1) Antenatal care;  2) Labour and delivery information;  3) Complications;  4) Postnatal care;  5) Outcome |
| 2018 | Save the Children^56^ | newborn | Registers, reporting forms | Not specified | 7 | 1) KMC initiation rate;  2) KMC referral completion;  3) Newborn survival to discharge;  4) Newborn death before discharge;  5) Left against medical advice. |
| 2020 | Bhattacharya^29^ | Maternal, newborn | Case notes, registers | Both | Varied between health facility | 1) First antenatal care visits;  2) Total antenatal visits;  3) Facility deliveries;  4) Antenatal care anaemia testing;  5) Antenatal care syphilis testing;  6) Iron-folic acid supplementation;  7) At least one dose administered of intermittent preventive treatment of malaria;  8) At least one dose administered of tetanus toxoid vaccine;  9) Delivery by skilled birth attendant;  10) Live birth or stillbirth;  11) Baby weighed at birth;  12) Oral polio vaccine given at birth;  13) Early postpartum-postnatal care within 3 days of birth;  14) BCG vaccine given during postnatal care period. |
| 1997 | Danquah^32^ | Maternal, newborn | Registers | Paper | Maternity roll: 20  Admission/ Discharge Register: 20 | 1) Registration information;  2) Demographics;  3) pregnancy information;  4) Dates (of admission, discharge, birth);  5) Delivery information and outcome;  6) Condition on discharge;  7) Complications and treatments given;  8) Infant information. |
| 2009 | Day^33^ | Newborn | Case notes | Paper | Demographics: 15  Problem list: 16 Admission 140 Discharge: 50  Daily progress: 50 | 1) Registration and demographics;  2) Admission history and examination;  3) delivery;  5)6) High risk fitting/ low glucose;  7) Discharge diagnosis sheet  8) Outcomes |
| 2009 | Day^34^ | Maternal, newborn | Case notes | Paper | Demographics: 15 Admission: 83  Delivery: 63 Infant assessment: 61  High risk: 40  Hypertensive disease: 23  Discharge diagnosis sheet: 99 | 1. Registration and demographics; 2. Antenatal care   3) Admission;  4) delivery;  5) infant assessment;  6) High risk previous caesarean/haemorrhage/twins/breech;  7) Hypertensive disease;  8) Discharge diagnosis sheet  9) Outcomes |
| 2010 | Day^35^ | Maternal, newborn | Case notes | Paper | Caesarean section: 97  Post-operative: 24 each day | 1) Caesarean section Operation note  2) Caesarean section summary indication/ post-op care  3) post-operative care |
| 2011 | Day^36^ | Maternal, newborn, child | Register / Case notes / interoperable system | Both | 62 | Not specified |
| 2020 | Day^37^ | Maternal, newborn | Registers | Paper | 21 | 1) Uterotonics for prevention of postpartum haemorrhage;  2) Breastfeeding early initiation in first hour after birth;  3) Baby resuscitation;  4) Antenatal Corticosteroid;  5) Chlorhexidine applied to cord;  6) Birth outcome and stats;  7) Birth weight;  8) Gestational age;  9) Woman’s outcome at discharge;  10) Baby’s outcome at discharge;  11) Mother demographics;  12) Retained placenta;  13) Estimated blood loss. |
| 2014 | Hawley^10^ | Maternal, newborn | Case notes | Both | 28 | 1) Clinical measurement;  2) Screening;  3) Pregnancy assessments/advice;  4) Immunisation – pre-conception assessment;  5) Immunizations required in pregnancy |
| 2023 | Kabue^39^ | Maternal, newborn | Registers | Both | Varied between health facilities; Range: 24 (Nepal) – 188 (Eswatini) | 1) Key measurements during antenatal care visits;  2) IPT doses, malaria treatment and bed nets;  3) Screening and testing performed during antenatal care visits;  4) Childbirth information;  5) Newborn care during the first hour of life;  6) Abortion, foetal and early death of the newborn;  7) HIV testing and treatment services. |
| 2016 | Kosgei^41^ | Maternal, newborn | Case notes | Not specified | Varied between health facilities | 1) Resource availability;  2) admission documentation;  3) Partograph details on admission;  4) Eclampsia documentation;  5) Complications |
| 2020 | Lodge^43^ | Maternal, newborn | Case notes | Paper | 18 | 1) Patient characteristics;  2) Patient progress and monitoring;  3) Surgical site infections/sepsis. |
| 2011 | Lugthart^44^ | Child | Case notes | Paper | TBC | Full IMCI: danger signs, main symptoms, anaemia, nutrition, immunizations, feeding |
| 2022 | Muinga^46^ | Newborn | Case notes | Paper | 74 | 1) Biodata;  2) Feed and fluid prescription;  3) Vitals and assessment monitoring;  4) Nursing shift notes;  5) Input (feed and fluid) balance. |
| 2022 | Pahlevanynejad^48^ | Maternal, newborn | Case notes | Not specified | 233 | 1) Demographic information;  2) Mother's before pregnancy information;  3) Mother's pregnancy information;  4) Information about the birth and post-birth hospital stay. |
| 2015 | Santos Alves^49^ | Maternal, newborn | Case notes | Digital | 179 (157 for the initial prenatal visit and 56 for subsequent visits) | 1) Data identification;  2) Socio-demographic variables;  3) Personal background;  4) Family backgrounds;  5) Gynaecological and obstetric backgrounds;  6) Current pregnancy;  7) Physical exam;  8) Obstetric physical exam;  9) General physical exam;  10) Clinical exam;  11) Labs and imaging;  12) Adherence to obstetrics guidelines. |
| 2019 | Tully^21^ | Newborn, child | Case notes | Both | 24 | 1) Breast feeding and solid food status;  2) Times per day/week the child eats/drinks breast milk, formula, cow's milk, juice or soda, other milks, eggs, meat, baby food, starchy vegetables, green vegetables, fruit, sweet snacks, finger foods, spooned foods, new foods;  3) How well the child tolerates food. |
| 2020 | Zakaria^25^ | Maternal, newborn | Registers | Digital | 256 | Not specified |

Abbreviations – BCG: Bacillus Calmette–Guérin; IMCI: Integrated management of childhood illness; IPT: intermittent preventive treatment; KMC: Kangaroo mother care
